# Use of the European standardization framework established by CEN/TC 216 for effective disinfection strategies in human medicine, veterinary medicine, food hygiene, industry, and domestic and institutional use – a review

**DOI:** 10.3205/dgkh000417

**Published:** 2022-07-07

**Authors:** Astrid Bolten, Verona Schmidt, Katrin Steinhauer

**Affiliations:** 1 BODE Chemie GmbH, Hamburg, Germany; 2 Chemische Fabrik Dr. Weigert GmbH & Co. KG, Hamburg, Germany; 3 bactologicum GmbH, Itzehoe, Germany; 4 Faculty of Mechanical Engineering, University of Applied Sciences, Kiel, Germany

**Keywords:** disinfection, human medicine, veterinary medicine, food, industry, EN 14885, norms, microbicidal efficacy, virucidal efficacy

## Abstract

The SARS-CoV-2 pandemic illustrates the necessity of effective preventive measures for existing and newly emerging pathogens. When confronted with pathogens or spoilage agents, especially if they are not yet well studied, effective hygiene protocols are needed immediately. In the medical field, effective preventive measures are key to prevent vulnerable patients from infections. In production areas, effective hygiene measures are needed to protect goods from spoilage or microbial contamination.

The European standardization framework established by the European Committee for Standardization (CEN) ensures that effective hygiene measures are available and can be immediately implemented when needed. Based on a broad portfolio of standards/laboratory tests, activity claims specifically addressing the special features of applications of antimicrobial formulations are substantiated. In this review, the concept of using standardized surrogate test organisms is explained, and the European standardized test approach to claim microbicidal and virucidal efficacy, the specificity of claims and their relevance for infection prevention measures is illustrated. Furthermore, relevance of the European Norm test methods is elucidated in the light of legal requirements.

Finally, the review explains the systematics of the standardized methodological portfolio of CEN, Technical Committee 216, which is very useful when effective strategies for fighting or preventing microbial and viral induced infections, contaminations or spoilage are needed on an immediate basis.

## Abstract (de)

Die SARS-CoV-2-Pandemie verdeutlicht die Notwendigkeit wirksamer Präventionsmaßnahmen für bekannte und neu auftretende Krankheitserreger. Wenn man mit Krankheits- oder Verderbniserregern konfrontiert wird, insbesondere wenn sie noch nicht gut erforscht sind, sind sofort wirksame Hygieneprotokolle erforderlich. Im medizinischen Bereich sind wirksame Präventionsmaßnahmen von entscheidender Bedeutung, um efährdete Patienten vor Infektionen zu schützen. Ebenso sind in Produktionsbereichen wirksame Hygienemaßnahmen erforderlich, um Waren vor Verderb oder mikrobieller Verunreinigung zu bewahren. Mit Hilfe der vom Europäischen Komitee für Normung (CEN) erarbeiteten Normen im Bereich der Desinfektion und Antisepsis können wirksame Hygienemaßnahmen kurzfristig und bedarfsgerecht etabliert werden. Auf der Grundlage eines breiten Portfolios von Normen/Labortests werden Wirksamkeitsauslobungen begründet, die speziell die Besonderheiten bei der Anwendung mikrobizider Formulierungen im Fokus haben. In der Übersichtsarbeit wird das Konzept der Verwendung standardisierter Surrogat-Testorganismen erläutert und das standardisierte europäische Methodenportfolio zur Auslobung der mikrobiziden und viruzider Wirksamkeit, die Spezifität der Auslobung und ihre Relevanz für Maßnahmen zur Infektionsprävention veranschaulicht sowie die Relevanz der Testmethoden der Europäischen Normen im Hinblick auf die gesetzlichen Anforderungen erläutert. Abschließend wird die Systematik des standardisierten Methodenportfolios des Technischen Komitees 216 des CEN erläutert, das von großem Nutzen ist, wenn wirksame Strategien zur Bekämpfung oder Verhinderung von mikrobiell oder viral bedingten Infektionen, Kontaminationen oder Verderb unmittelbar erforderlich sind.

## Background

In December 2019, the city of Wuhan in China became the center of an outbreak of a novel coronavirus. Investigation of the SARS CoV-2 revealed a similarity of at least 70% to the genetic sequence of the SARS CoV (severe acute respiratory syndrome-related coronavirus), which emerged in 2002 in China and spread around the world [1]. Coronaviruses, enveloped single-stranded RNA viruses, are characterized by club-shaped spikes on the surface of the virion, prompting the name coronavirus due to the similarity in appearance to a solar corona [2]. Until the SARS-CoV outbreak in 2002, coronaviruses were thought to only cause mild self-limiting infections in humans but were known to cause a wide variety of infections in animals [2]. In order to contain human infections, in many cases, only limited preventive measures such as vaccination or treatment with therapeutic antibodies are available. 

In order to prevent spreading, the WHO recommended hygiene measures such as use of 70% ethanol as a disinfectant [3]. To also enable the use of other suitable disinfectants, the German Robert Koch Institute already recommended the use of disinfectants claiming “limited virucidal activity”, which includes only enveloped viruses, during the SARS CoV outbreak, which is also valid in the context of the current SARS CoV-2 outbreak [4], [5].

This recommendation is based on the methodological framework of the European Committee for Standardization (CEN). CEN has defined a set of surrogate test organisms which are representative for certain groups of microorganisms. A proven efficacy against these representative surrogate test organisms allows efficacy claims for the respective group of organisms, e.g., bactericidal, yeasticidal, fungicidal or virucidal efficacy [6]. For the claim “virucidal activity against enveloped viruses”, MVA (modified vaccinia virus Ankara) has been defined as the suitable surrogate organism [7], [8]. 

The current SARS CoV-2 pandemic situation thus demonstrates the importance of standardized test protocols to evaluate chemical disinfectants and antiseptics on a superordinate basis. The use of a defined set of standard surrogate test organisms helps to choose effective disinfectants for a specific outbreak or infection prevention scenario without the rather time consuming need to test each contagious agent individually. Thus, EN (European norm) methods from CEN/TC 216 ensure that effective hygiene measures are put in place on an ad hoc basis.

European standards from CEN/TC 216 are significant in the field of infection prevention and disinfection in Europe and are also in demand in the US, Canada or the Asian Pacific Area.

This review aims to elucidate the systematic approach of the European standards established by the CEN/TC 216 and provide guidance on choosing suitable chemical disinfectants based on the expertise of CEN/TC 216. The European standardized test approach to claim antimicrobial and virucidal efficacy, the use of surrogate test organisms, the specificity of claims and their relevance for infection prevention measures under real conditions are explained. Furthermore, relevance of the EN test methods is explained in the light of legal requirements.

## Scope and structure of CEN/TC 216

The standardization activities of CEN are steered by the CEN Technical Board (BT), which has full responsibility for the execution of CEN's work programme. Standards are prepared by **Technical Committees** (TCs). Each TC has its own field of operation (scope) within which a work program of identified standards is developed and executed. The scope of the **CEN/TC** **216** is standardization of the terminology, requirements, test methods including potential efficacy under in-use conditions, recommendations for use and labelling in the whole field of chemical disinfection and antiseptics. Areas of activity include agriculture (but not crop protection chemicals), domestic service, food hygiene and other industrial fields, institutional, medical and veterinary applications [9]. 

The standards development is undertaken by **Working Groups** (WGs) where experts, appointed by the CEN Members, come together and draw up a draft that will become the future standard. This reflects an embedded principle of 'direct participation' in the standardization activities.

This is reflected by the structure of CEN/TC 216 “chemical disinfectants and antiseptics,” which comprises 4 different working groups, each of them concentrating on a specific field of application. Experts from academia, public authorities, laboratories, and industry work together to provide guidance on test protocols and requirements for effective chemical disinfectants in the fields of human medicine, veterinary use, food hygiene, and domestic and institutional use [10]. 

**Figure 1:**
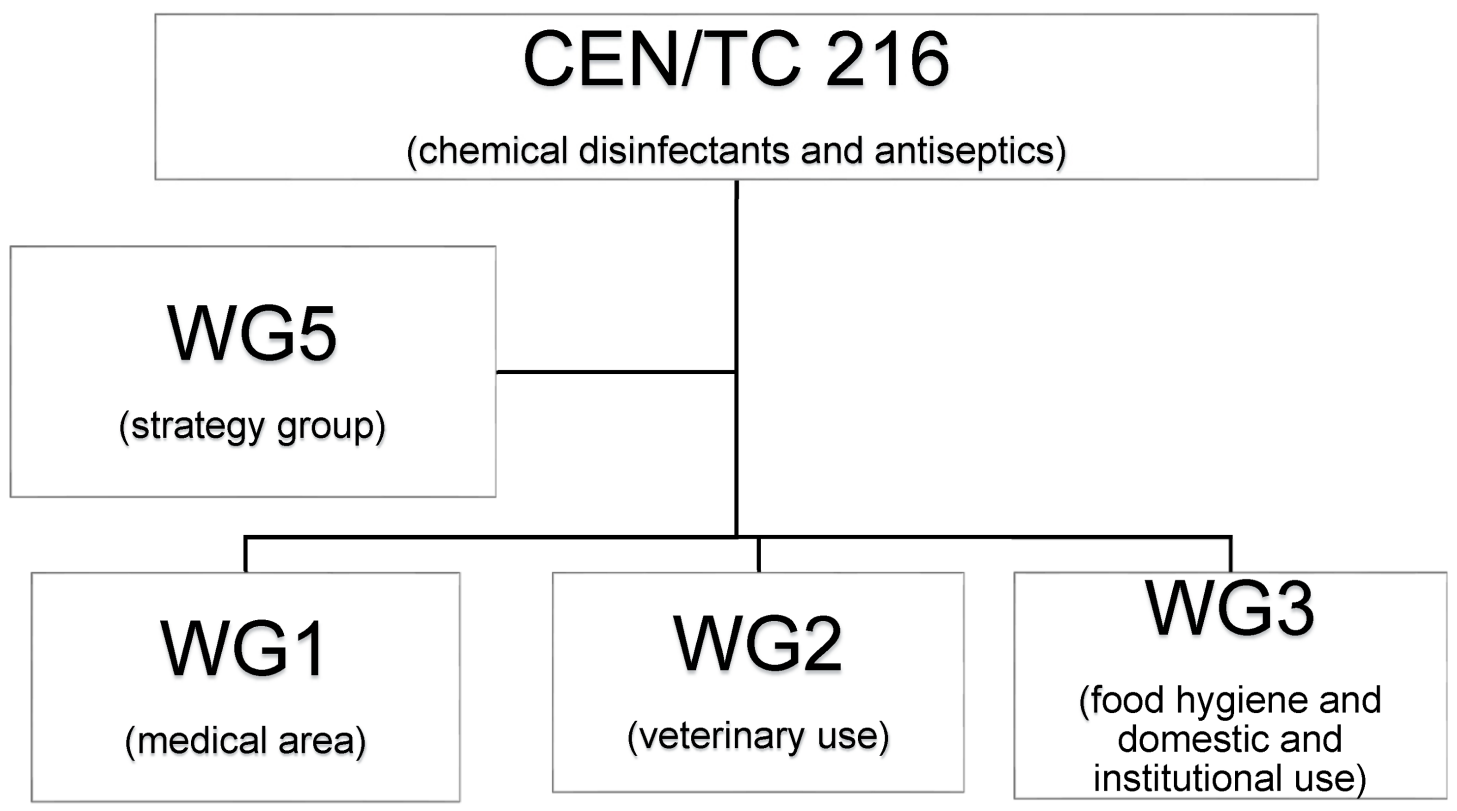
Structure of CEN/TC 216 and the different working groups

Within CEN/TC, 216 different working groups are established to define standardized test methods and requirements for the antimicrobial efficacy of chemical disinfectants and antiseptics (Figure 1 (Fig. 1)). The focus of WG 1, human medicine, is intended to prevent infections of humans, where disinfection or antiseptics are medically indicated. The focus of WG 2 – veterinary use – is intended to prevent the transmission of infection in the veterinary field. This include products used in the breeding, husbandry, production, veterinary care facilities, transport and disposal of all animals except when in the food chain following death and entry into processing industry. The focus of WG 3 – food hygiene and domestic and institutional use – is intended to prevent the transmission of infection in food hygiene, industry, and domestic and institutional fields, excluding areas and situations where disinfection is medically indicated and/or used on living tissues, except those for hand hygiene in the fields mentioned above. The scope applies at least to the following: processing, distribution and retailing of food of animal origin, food of vegetable origin; institutional and domestic areas and other industrial areas.

Additionally, working group 5 (WG 5) covers all transversal and strategic aspects, which is reflected in the transversal standards EN 14885 and EN 12353 [6], [11]. These standards were prepared by the Technical Committee CEN/TC 216 “Chemical disinfectants and antiseptics” and shall be given the status of a national standard.

In EN 14885, all general aspects for the different fields of application, including the requirements of the respective standards from WG 1, WG 2, and WG 3, are summarized and published on a regular basis. 

The first edition of EN 14885 was published in 2007 and has been updated ever since on a regular basis to reflect the latest developments of standardization in CEN/TC 216. 

EN 14885 specifies the requirements for activity testing of chemical disinfectants and antiseptics to support antimicrobial activity claims of disinfectant products. In doing so, EN 14885 aims to

- enable manufacturers of chemical disinfectant products to choose the appropriate standards/laboratory methods to support efficacy claims,
- enable users of chemical disinfectant products to assess the information provided by the manufacturer,
- support regulatory authorities in assessing claims made by manufacturers of disinfectant products.

The standard is applicable to products to be used in the area of human medicine (WG 1), veterinary area (WG 2) and in food hygiene, industry, and domestic and institutional areas (WG 3). For all these areas, standards/laboratory tests are available to test products and support activity claims specifically addressing the special features of each area of application.

## Relevance of EN test methods in the light of legal requirements

The work of CEN/TC 216 is also highly relevant considering the legal aspects for medical devices, biocides and drugs, as disinfectants approved for the market are regulated under these legal categories in Europe. Thus, some of the European standards developed by CEN/TC 216 are developed under a mandate from the European Commission (EC) and European Free Trade Association (EFTA). Those standards provide the basis for the technical documentation requested for medical devices as required by the European Medical Devices Regulation [12] to legally ensure safety and performance as claimed [12]. In this context, EN 14885 as the superordinate standard is referred to as a “harmonized standard” and is thus regarded an essential requirement within the authorization procedure for medical devices. As the category “medical device” applies only to disinfectants used in healthcare, the standards developed by the experts from WG 1 in CEN/TC 216, which are referred to in the superordinate standard EN 14885, are relevantin in terms of the European Medical Devices Regulation [12]. Thus, both chemical disinfectants for instrument disinfection in healthcare and antiseptics need to be evaluated in a conformity assessment based on the European Medical Devices Regulation [12] and are registered as medical devices. The conformity assessment is directly linked to the relevant standards referred to in the harmonized standard EN 14885 and those are mandatory for the assessment. 

Furthermore, chemical disinfectants for all other fields of application (except for disinfectants classified as medical devices as mentioned above) in Europe must be registered under the Biocidal Products Regulation [13] as biocides. Registration as a biocide may be exclusive (e.g., hand disinfectants) or in addition to a legal registration under MDR [12]. Thus, some chemical disinfectants may be registered as a medical device as well as a biocide and are so-called “dual-use” products. Surface disinfectants are an example of “dual-use” products , which in the patient’s immediate surroundings are used as medical devices themselves to disinfect other medical devices, such as incubators, and therefore must be registered under MDR [12]. At the same time, the identical disinfectant may be used to disinfect the floor in the patient’s room, which is regarded a biocidal use for which a registration under BPR [13] is mandatory. Thus, surface disinfectants in Europe used in the medical field usually fall under the BPR [13] and the MDR [12] legislation.

In this context, it is important to note that whereas the European Medical Devices Regulation (MDR) [12] targets patient safety in terms of infection prevention, the Biocidal Products Regulation (BPR) [13] instead targets toxicological and environmental aspects to protect living beings and the environment from a potential chemical hazard. Both regulations evaluate the microbicidal/virucidal efficacy of chemical disinfectants and antiseptics based on the methodological framework established by CEN/TC 216. Under MDR [12], a direct link exists as described above by using a “harmonized standard” resulting in a mandatory application of European standards as published by CEN/TC 216. However, under BPR [13], some modifications of the methods have been requested by the European Chemical Agency (ECHA) as published in their guidance documents [14], [15]. Despite these discrepancies, a recently established exchange between EChA and CEN is intended to facilitate harmonization between EChA and CEN requirements when using the European standards developed by CEN/TC 216.

## EN methods – testing principles and procedures

The testing of efficacy within the methodological framework of CEN/TC 216 is performed according to a 3-phase graded test hierarchy: 

### Phase 1 tests

Quantitative suspension tests examine whether an active substance or product under development has bactericidal, fungicidal or sporicidal activity without regard to specific areas of application. As phase 1 tests are only for products in development, they cannot be used for any activity claim. Three phase 1 tests exist to date: EN 1040 [16] for bactericidal activity, EN 1275 [17] for yeasticidal and fungicidal activity and EN 14347 [18] for sporicidal activity.

### Phase 2 tests

These are designed for different application areas, e.g., human medicine, veterinary medicine, food hygiene, industry, domestic and institutional areas, and also for different intended uses, e.g, hand rub, surface disinfection, or instrument disinfection. The aim of phase 2 tests is to simulate practical use conditions for the intended application area in the best possible way. They comprise two steps. Phase 2/step 1 tests are quantitative suspension tests to establish that a product has bactericidal, fungicidal, yeasticidal, mycobactericidal, tuberculocidal, sporicidal or virucidal activity under simulated practical conditions appropriate to its intended use. Phase 2/step 2 tests are quantitative laboratory tests to establish that a product has bactericidal, fungicidal, yeasticidal, mycobactericidal, tuberculocidal or virucidal activity when applied to a specific carrier under simulated use conditions (so-called “carrier tests”) to mimic the practical use. Carriers used in phase 2/step 2 tests comprise surfaces simulating practical conditions, for instance, surface or instrument disinfection. Phase 2/step 2 tests for the evaluation of handwash or hand rub formulations are based on volunteer tests.

As an example, the quantitative carrier tests for instruments in human medicine simulate the immersion of medical instruments, and contamination is simulated with a dried inoculum in the presence of organic soiling, such as bovine serum albumin (BSA) and sheep erythrocytes [19], [20], [21], [22]. Another example is testing of surface disinfectants. Chemical disinfectants that are used for surface disinfection are often applied by wiping. Thus, an appropriate test procedure should also mimick the mechanical action (wiping) when testing surface disinfectants, which is realized in the 4-field test [23]. In this test model, wiping is simulated by using a standardized weight on defined non-porous test surfaces [23], [24].

Within the methods of CEN/TC 216, the tests differ in terms of application area. For instance, test organisms, soiling, temperature and surfaces differ depending on whether the disinfectant is intended to be used in human medicine, veterinary medicine, food hygiene, industry, or domestic and institutional areas, as each field has specific requirements. Therefore, pass criteria, organic loading, contact time, test temperatures, test organisms and the procedure of quantitative carrier tests (phase 2, step 2 test) have been designed to reflect practical conditions of the different application areas and different use scenarios.

For example, in the food industry, disinfectants may also be used in cold rooms. Therefore, the corresponding standards provide test temperatures of 4°C or 10°C in addition to room temperature. In contrast, products that are used at temperatures ≥40°C, which is the case in textile disinfection, bactericidal activity has to be demonstrated using the Gram-positive thermostable bacterium *Enterococcus faecium* [25], [26], [27] as a test organism.

Another example is the use of bacteriophages as test organisms for efficacy tests in the food, industrial, domestic and institutional areas. As bacteriophages are known contaminants in numerous biotechnological production or fermentation processes [28], chemical disinfectants used in these applications must be tested against two species of *Lactococcus lactis* subsp. lactis bacteriophages (bacteriophage P001 and P008) in the presence of skimmed milk and sour whey [29]. For example, effective biocidal formulations can thus be chosen to prevent lysis due to phage contamination of bacterial starter cultures used in cheese production .

### Phase 3 tests

 In addition to the existing phase 2/step 2 tests mimicking varying application conditions, in the future, phase 3 tests could complete the methodological framework. Phase 3 tests are field tests under real practical conditions, and may be used to validate efficacy of disinfection protocols more specifically under real conditions, if necessary. However, at the moment, applicable methodologies for this type of test are not yet available, but are intended to be developed in the future. 

## EN methods – test organisms

All standards relate to a defined range of microbial species, which are summarized as an example of the CEN/TC 216 quantitative suspension tests (phase 2/step 1) in Table 1 (Tab. 1).

These test organisms have been chosen as representative species considering their relative resistance, relevance to practical use in the respective area of application, and handling properties, including microbiological laboratory safety aspects. To claim antimicrobial efficacy of disinfectants, a defined set of surrogate organisms which allow claims to be made for bactericidal, yeasticidal, fungicidal and virucidal efficacy, etc. is used. For example, *Candida albicans* has been defined as a suitable surrogate test organism for claiming yeasticidal efficacy. Thus, disinfectants claiming yeasticidal efficacy based on EN standards also cover other yeasts, such as the human pathogen *Candida auris*, when applying the use recommendations made for yeasticidal efficacy [30]. Likewise, in the SARS-CoV-2 pandemic, effective disinfection measures were needed immediately. As a coronavirus, SARS-CoV-2 is an enveloped virus. Thus, the methodological framework of CEN/TC 216 helps to identify effective formulations based on the claim ‘virucidal activity against enveloped viruses’ on an immediate basis[6], [8], [31], [22]. In the EN methods, the claim ‘virucidal activity against enveloped viruses’ is secured by experimental standardized tests using vaccina virus, which has been demonstrated to be an appropriate surrogate virus [7], [32]. Several studies prove the EN claim ‘virucidal activity against enveloped viruses’ to cover numerous viral human pathogens belonging to the enveloped viruses, such as MERS CoV, SARS CoV, Zika virus or Ebola virus [7], [32], [33]. In the medical area (WG 1), spores of *Clostridium difficile* R027 are used to claim activity against *Clostridium difficile* [34]. This unusually specific claim within CEN/TC 216 reflects the specific relevance of* C. difficile* in the clinical setting. Numerous reports of *C. difficile* outbreaks exist, emphasizing that *C. difficile* is an important pathogen worldwide [35]. CEN/TC 216 reacted to this specific need to provide healthcare facilities with sporicides which have been tested under standardized test conditions, specifically targeting spores of *C. difficile* by establishing EN 17126 [34], [36]. 

Thus, based on the concept of using test organisms representative of wider groups to claim microbicidal efficacies such as ‘bactericidal’, ‘yeasticidal’, ‘sporicidal’, ‘virucidal’ or ‘virucidal activity against enveloped viruses’, effective disinfectants are available immediately. 

The concept of using a defined set of microorganisms representative of wider groups also includes efficacy claims for multidrug-resistant organisms (MDRO). Several studies investigated the sensitivity of MDRO in comparison to non-resistant reference strains, and compared the activity of biocidal agents against the tested strains, including fungi and bacteria. Activity of biocidally agents against MDRO was found to be at least equal when compared to the non-resistant reference strains [37], [38], [39], [40]. Thus, based on the methodological framework of CEN/TC 216, antimicrobial efficacy of disinfectants also covers efficacy against MDRO due to the defined set of surrogate organisms, and allows immediate implementation of appropriate disinfection procedures. 

## EN methods – organic soiling mimicking practical conditions

The efficacy of disinfectants may be influenced by organic matter, as has been described in several studies [41], [42], [43]. EN test methods thus take this phenomenon into consideration and, in order to simulate the practical use of biocidal formulations, tests are carried out using different soiling conditions that mimick practical conditions. In their study of organic contamination found under real conditions in comparison to the artificial soiling used in the EN test methods, Meyer et. al. [44] investigated the amount of soiling in a professional kitchen, demonstrating that the soiling defined in the EN test methods represents the worst-case soiling found in practice.

Soiling is usually simulated using bovine serum albumin (BSA) and/or erythrocytes from sheep blood. The general soiling levels are ‘clean conditions’ (i.e., low-level soiling) and ‘dirty conditions’ (i.e., high-level soiling). Clean test conditions are representative of surfaces which have been cleaned satisfactorily and/or are known to contain only minimal levels of organic and/or inorganic substances. In the veterinary area, these conditions are called “low-level soiling”, because the respective levels of soiling used in the veterinary test methods are higher, due to higher soiling levels generally expected in practice in the veterinary field. Dirty test conditions are representative of surfaces which are known to or may contain organic and/or inorganic substances. In the veterinary area, these conditions are called “high-level soiling”, because the respective levels of soiling in practice are considered higher.

In the medical area, formulations that are tested under dirty conditions, e.g., hygienic handwash products or surface disinfectants, must demonstrate effectiveness in the presence of a combination of proteins (bovine serum albumin) and blood erythrocytes from sheep blood. For disinfectants used in food, industrial, domestic and institutional areas, organic loading is only based on bovine serum albumin (BSA). 

Within the methods of CEN/TC 216, other soiling conditions mimicking specific application areas may also be used. These additional soiling conditions are described in the respective standards for the different application areas. As an example for the use in dairies, skimmed milk may be used, and in breweries, yeast extract may be used. This takes more specific conditions found in practice into account and secures claims for product use under these specific conditions as well.

### Kinetic test range to secure data validity

Within the EN standards of CEN/TC 216, testing of disinfectants has been established in most standards in a manner that allows efficacy kinetics to be demonstrated within the tests to secure data validity. Thus, per contact time, product test solutions are prepared at a minimum of three different concentrations, including one concentration in the active range and one concentration in the non-active range. The number of surviving test organisms in each sample is determined and the logarithmic reduction factor is calculated based on the surviving test organisms in each respective sample. This enables evaluation of the the mode of action kinetics of the respective biocidal formulations to be tested. Additionally, this test scheme takes environmental, occupational health and economic aspects into account by ensuring that products at their use concentration do not contain more active substances than necessary, while still being safe in their given microbicidal application [6]. 

### Requirements for microbicidal claims

Antimicrobial efficacy in EN standards is determined on the basis of decadic logarithmic reduction (lg). In the experimental set up of EN methods, a defined inoculum of test organisms is used and lg reduction of test organisms by the tested formulation is determined. Based on the area of application (human medicine [WG 1], veterinary area [WG 2] or food hygiene, industry, domestic and institutional areas [WG 3]) each claim (e.g., bactericidal, yeasticidal, sporicidal, etc.) is based on a specific lg reduction requirement (Table 2 (Tab. 2), Table 3 (Tab. 3), Table 4 (Tab. 4), Table 5 (Tab. 5), Table 6 (Tab. 6), Table 7 (Tab. 7)). 

The lg reduction factor requirement is recorded in each standard of CEN/TC 216 and is summarized in EN 14885 [6]. Thus, for example, a bactericidal claim for surface disinfection without mechanical action in the human medicine area requires a lg reduction ≥5.0 to be passed in both the quantitative suspension test an the quantitative carrier test, as recorded in EN 13727 (phase 2/step 1 test) or EN 17387 (phase 2/step 2 test) [26], [45]. In the food, industrial, domestic and institutional areas, bactericidal efficacy requires a lg reduction ≥5.0 to be passed in the quantitative suspension test EN 1276 [46] (phase 2/step 1 test) and a lg reduction ≥4.0 to be passed in the quantitative carrier test EN 13697 (phase 2/step 2 test) [47]. In this context, the more stringent requirements in the human medicine area than in the food, industrial, domestic and institutional areas are based on the rationale that in human medicine, vulnerable or even immunosuppressed patients must be protected from transmission of microbial infections. In contrast, in the food, industrial, domestic and institutional areas, use of disinfectants is not medically indicated, and people can be expected to be immunocompetent. As a result, the pass criteria for microbicidal claims for disinfectants used in the human medicine area are higher than in the food, industrial, domestic and institutional areas. 

## Future developments of EN methods

Future developments of EN methods aim to reflect current topics in hygiene. In recent years, a significant increase in *Clostridiodes difficile* acquired diarrhoea (CDAD) in the medical area has been identified [35]. In this context, surface disinfection has been described as an effective measure to control transmission of *C. difficile* via inanimate surfaces [48]. CEN/TC WG 1 takes this important topic into consideration by developing a phase 2/step 2 carrier test mimicking surface disinfection for testing sporicidal chemical disinfectants applied by wiping. The methodology is based on EN 16615 [23], and the robustness of the test protocol has been evaluated by ring trial experiments [49]. Likewise, a standardized hand rub protocol to test the efficacy of hand rubs against murine norovirus as a test virus is under development, and has already been pre-evaluated in ring trial experiments and published as prEN 17430 [50], [51].

In the veterinary area, a standardized test is under development mimicking teat disinfection. In this test method, artificial skin is used as a standardized carrier in a quantitative carrier test (phase 2/step 2; [52]).

In the food, industrial, domestic and institutional areas, future method developments are, for example, addressing the evaluation of residual antimicrobial (bactericidal and/or yeasticidal) efficacy of liquid chemical disinfectants on hard non-porous surfaces in a quantitative carrier test (phase 2/step 2). Additionally, a test method to evaluate microbicidal efficacy of surface disinfectants applied by wiping is being worked on (quantitative carrier test; phase 2/step 2). Suitable test methods for the standardized evaluation of virucidal efficacy for food, industrial, domestic and institutional applications are under development as quantitative suspension and carriers tests, respectively. 

Last but not least, the experts from CEN/TC 216 review published methods on a regular basis, and if necessary, revise methods to reflect on state of the art science and technology. Thus, the European standardization framework established by CEN/TC 216 provides state-of-the-art methods to select effective disinfection strategies in human medicine, veterinaryuse , food hygiene, industry, and domestic and institutional areas.

## Conclusions

When effective disinfection protocols are needed, the methodological framework of CEN/TC 216 provides a solid basis to select effective formulations for a given prevention strategy. In case of the ongoing SARS-CoV-2 pandemic, disinfectants claiming ‘virucidal activity against enveloped viruses’ based on EN-methods [6], [8], [31] provide a safe basis for immediately choosing effective disinfectants against SARS-CoV-2. 

Based on the standardized microbicidal claims of EN methods, effective disinfectants can be selected immediately and without the need to establish rather time-consuming and costly new testing protocols for newly emerging pathogens such as SARS CoV-2. Efficacy claims based on EN methods will provide the user with relevant information for application. These efficacy claims consider the microbicidal efficacy spectrum, as well as cleanliness of surfaces , appropriate contact time and effective concentration.

The choice of effective disinfection protocols will also help to secure hygienic safety in production processes. If, for example, bacterial contaminants such as *Streptococcus* spp., *Staphylococcus* spp. or *Salmonella* spp. have been identified in hygiene controls in food production processes, disinfectants claiming bactericidal efficacy based on WG-3 methods (e.g. EN 1276 and EN 13697 [42], [43]) are an appropriate choice and should be applied according to the bactericidal claim in terms of concentration, contact time and soiling conditions.

In the veterinary area, chemical disinfectants and antiseptics are to be used in the areas of breeding, husbandry, veterinary care facilities, production, transport and disposal of animals and veterinary laboratories for analyses and research [6]. In case of fighting viral infections such as foot-and-mouth disease (FMD) in the veterinary area, effective disinfectants have to be tested according to EN 14675 [53] against the non-enveloped double-stranded RNA virus enteric cytopathic bovine orphan virus type 1 (ECBO) [54]. Enteroviruses as well as the viruses causing foot-and-mouth disease (FMD) belong to the Picornaviridae family [55]. Thus, chemical disinfectants claiming virucidal activity based on EN 14675 [53] will be an effective choice when fighting not only foot-and-mouth disease (FMD).FMD is a viral disease affecting even-toed ungulates such as bovids, and can cause severe or fatal central nervous system diseases.

These examples underline the value of the standardized methodological portfolio of CEN/TC 216 to choose effective chemical disinfectants and antiseptics when fighting or preventing microbial induced infections, contaminations or spoilage.

## Notes

### Competing interests

Author AB is an employee of Bode Chemie GmbH, Hamburg, Germany. Author VS is an employee of Chemische Fabrik Dr. Weigert GmbH & Co. KG, Hamburg, Germany. Author KS is an employee of bactologicum GmbH, Itzehoe, Germany, and formerly was an employee of Schülke & Mayr GmbH, Norderstedt, Germany. 

## Figures and Tables

**Table 1:**
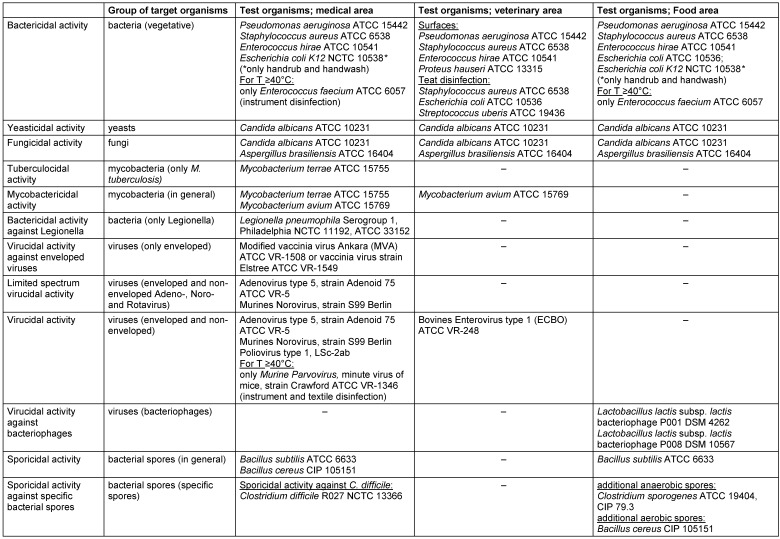
Summary of test organisms used in CEN/TC 216 quantitative suspension tests (phase 2, step 1)

**Table 2:**
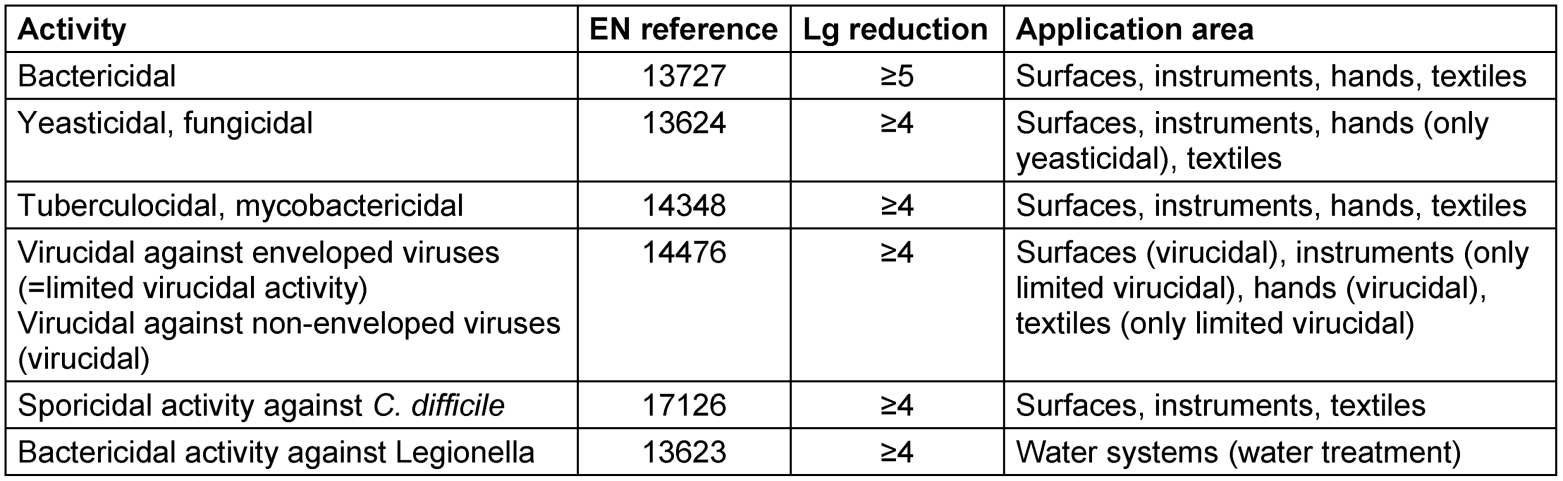
Quantitative suspension tests (phase 2, step 1) for the medical area

**Table 3:**
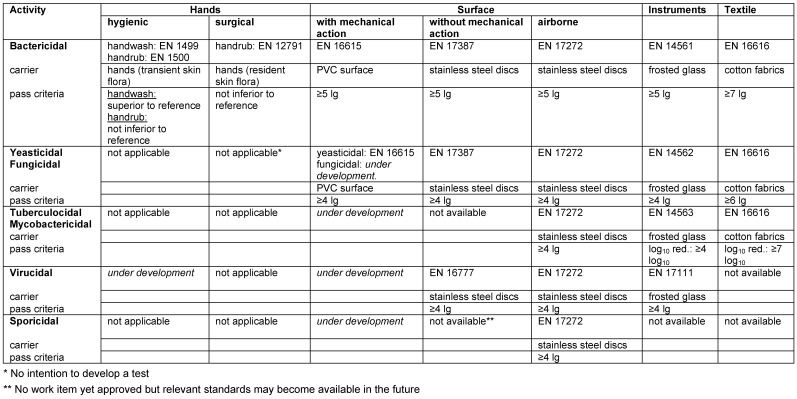
Quantitative carrier tests (phase 2, step 2) for the medical area

**Table 4:**
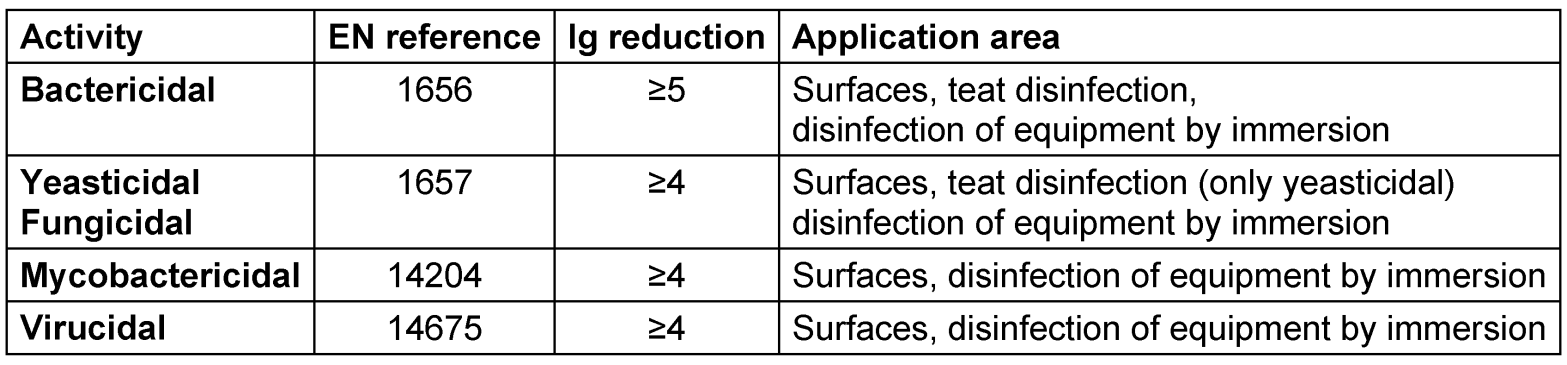
Quantitative suspension tests (phase 2, step 1) for the veterinary area

**Table 5:**
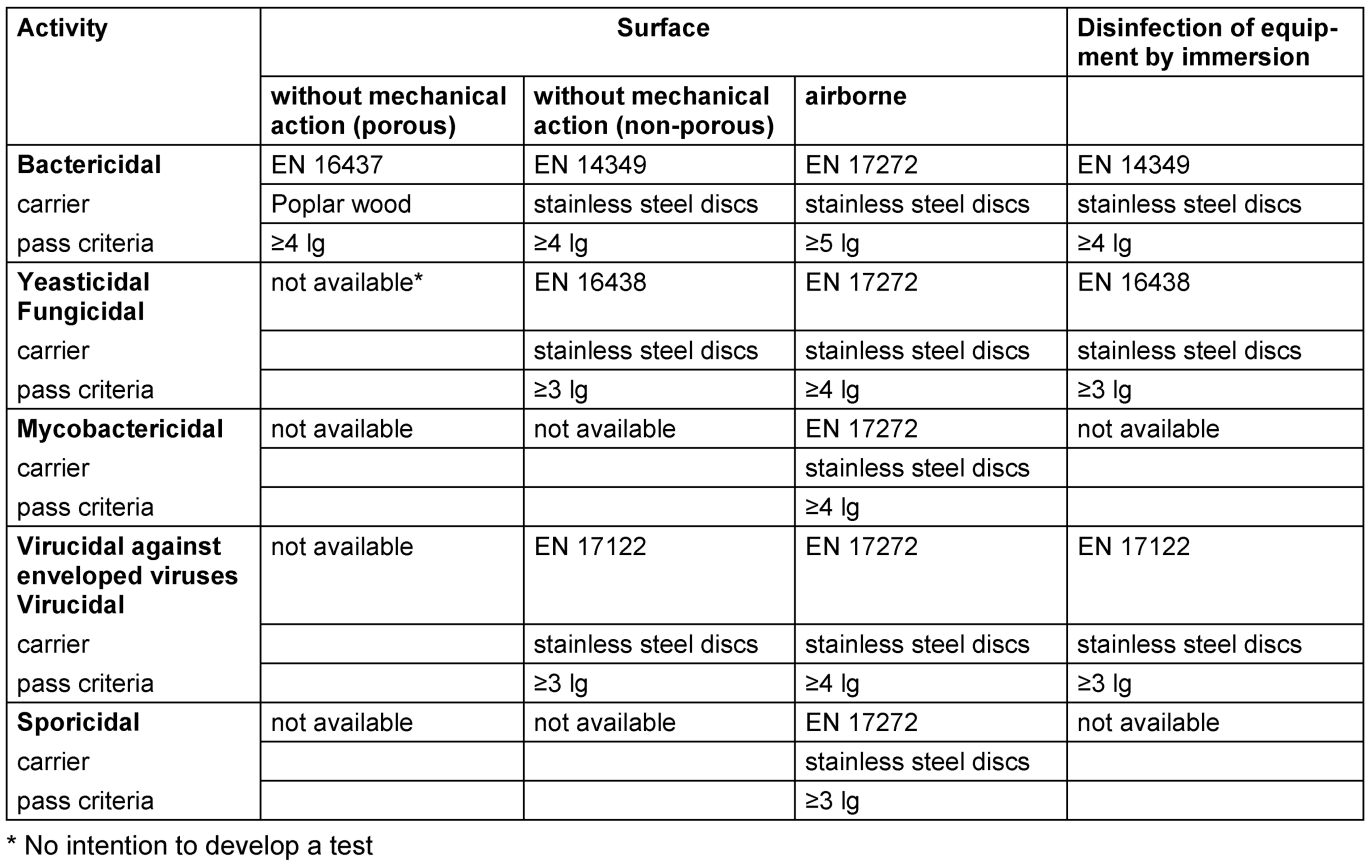
Quantitative carrier tests (phase 2, step 2) for the veterinary area

**Table 6:**
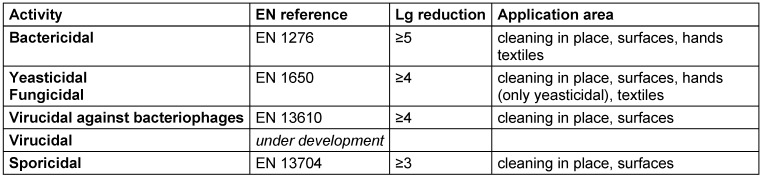
Quantitative suspension tests (phase 2, step 1) for the food, industrial, domestic and institutional areas

**Table 7:**
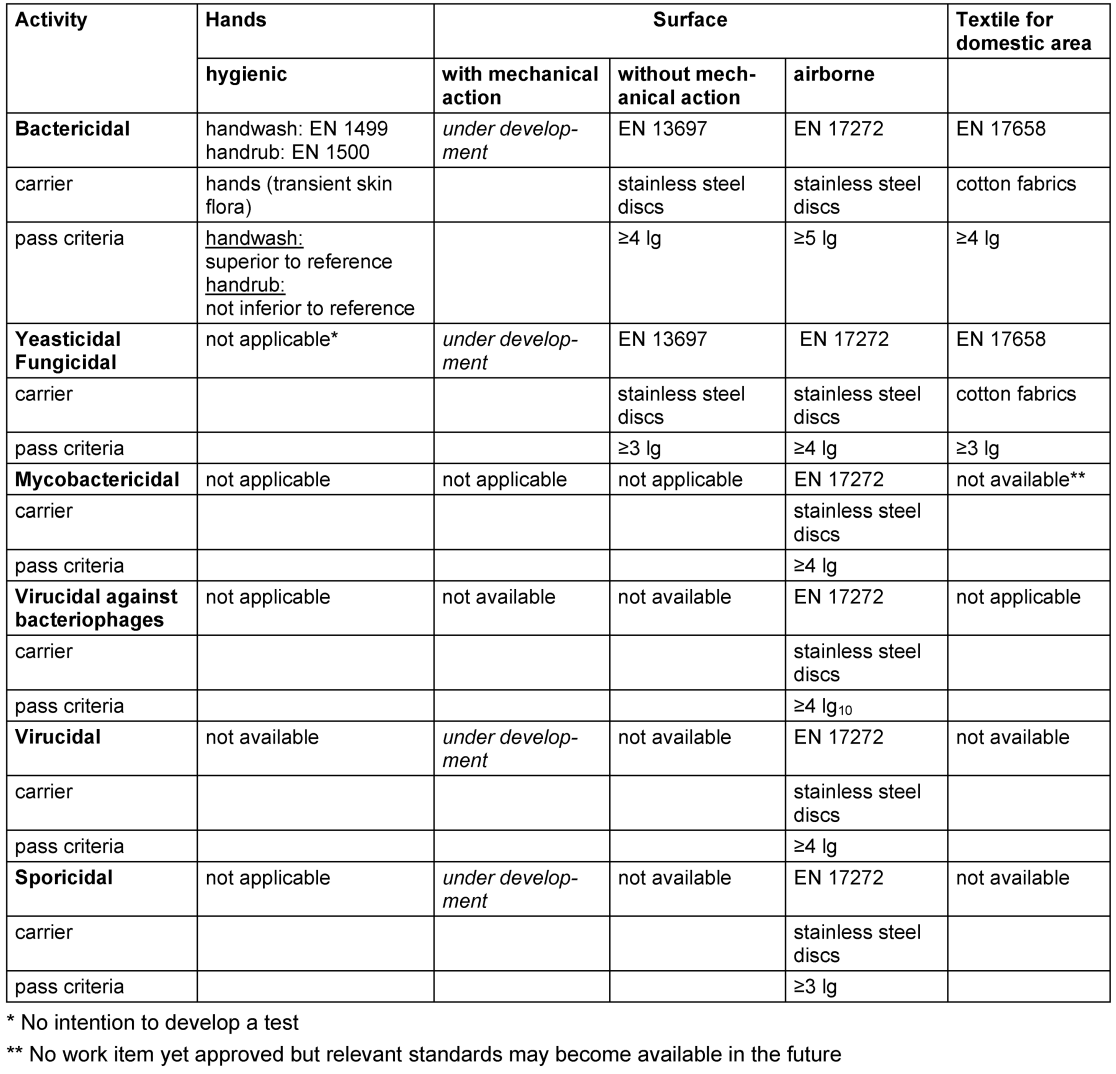
Quantitative carrier tests (phase 2, step 2) for the food, industrial, domestic and institutional areas

## References

[1] Hui DS, I Azhar E, Madani TA, Ntoumi F, Kock R, Dar O, Ippolito G, Mchugh TD, Memish ZA, Drosten C, Zumla A, Petersen E (2020). The continuing 2019-nCoV epidemic threat of novel coronaviruses to global health – The latest 2019 novel coronavirus outbreak in Wuhan, China. Int J Infect Dis.

[2] Fehr AR, Perlman S (2015). Coronaviruses: an overview of their replication and pathogenesis. Methods Mol Biol.

[3] World Health Organization (WHO) (2020). IInfection prevention and control during health care when novel coronavirus (nCoV) infection is suspected. Interim guidance.

[4] Robert Koch-Institut (RKI) (2020). Empfehlungen des RKI für die Hygienemaßnahmen und Infektionskontrolle bei Patienten mit Pneumonien verursacht durch ein neuartiges Coronavirus (nCoV) aus Wuhan, China.

[5] Robert Koch-Institut (RKI) (2015). Empfehlungen des Robert Koch-Institutes für die Hygienemaßnahmen und Infektionskontrolle bei Patienten mit Schwerem Akutem Respiratorischem Syndrom (SARS).

[6] European Committee for Standardization (2018). European Standard EN 14885: Chemical disinfectants and antiseptics – Application of European Standards for chemical disinfectants and antiseptics.

[7] Eggers M, Schwebke I, Suchomel M, Fotheringham V, Gebel J, Meyer B, Morace G, Roedger HJ, Roques C, Visa P, Steinhauer K (2021). The European tiered approach for virucidal efficacy testing – rationale for rapidly selecting disinfectants against emerging and re-emerging viral diseases. Euro Surveill.

[8] European Committee for Standardization (2019). European Standard EN 14476: Chemical disinfectants and antiseptics – Quantitative suspension test for the evaluation of virucidal activity in the medical area – Test method and requirements (Phase 2/Step 1).

[9] European Committee for Standardization https://standards.cencenelec.eu/dyn/www/f?p=205:7:0::::FSP_ORG_ID:6197&cs=10A8D4C3DCD7472E41B4ECBDD9E3C6A82.

[10] European Committee for Standardization https://standards.cencenelec.eu/dyn/www/f?p=205:29:0::::FSP_ORG_ID,FSP_LANG_ID:6197,25&cs=17E2B30DF09AC0FA861986AA85042A41B#1.

[11] European Committee for Standardization (2019). prEN 12353:2019 Preservation of test organisms used for the determination of bacterial (including Legionella), mycobacterial, sporicidal, fungicidal and virucidal (including bacteriophages) activity.

[12] European Union (2017). Regulation (EU) 2017/745 of the European Parliament and of the Council of 5 April 2017 on medical devices, amending Directive 2001/83/EC, Regulation (EC) No. 178/2002 and Regulation (EC) No. 1223/2009 and repealing Council Directives 90/385/EEC and 93/42/EEC. Off J Eur Union.

[13] European Union (2012). Regulation (EU) No 528/2012 of the European Parliament and of the Council of 22 May 2012 concerning the making available on the market and use of biocidal products. Off J Eur Union.

[14] European Chemicals Agency (EChA) (2018). Guidance on the BPR: Volume II Efficacy (Part A) – Version 2.0.

[15] European Chemicals Agency (EChA) (2018). Guidance on the BPR: Volume II Efficacy, Assessment + Evaluation (Parts B+C) – Version Version 2.0.

[16] European Committee for Standardization (2005). EN 1040:2005 Chemical disinfectants and antiseptics – Quantitative suspension test for the evaluation of basic bactericidal activity of chemical disinfectants and antiseptics – Test method and requirements (phase 1).

[17] European Committee for Standardization (2005). EN 1275:2005 Chemical disinfectants and antiseptics – Quantitative suspension test for the evaluation of basic fungicidal or basic yeasticidal activity of chemical disinfectants and antiseptics – Test method and requirements (phase 1).

[18] European Committee for Standardization (2005). EN 14347:2005 Chemical disinfectants and antiseptics – Basic sporicidal activity – Test method and requirements (phase 1).

[19] European Committee for Standardization (2006). EN 14561:2006 Chemical disinfectants and antiseptics – Quantitative carrier test for the evaluation of bactericidal activity for instruments used in the medical area – Test method and requirements (phase 2, step 2).

[20] European Committee for Standardization (2006). EN 14562:2006 Chemical disinfectants and antiseptics – Quantitative carrier test for the evaluation of fungicidal or yeasticidal activity for instruments used in the medical area – Test method and requirements (phase 2, step 2).

[21] European Committee for Standardization (2008). EN 14563:2008 Chemical disinfectants and antiseptics – Quantitative carrier test for the evaluation of mycobactericidal or tuberculocidal activity of chemical disinfectants used for instruments in the medical area – Test method and requirements (phase 2, step 2).

[22] European Committee for Standardization (2018). EN 17111:2018 Chemical disinfectants and antiseptics – Quantitative carrier test for the evaluation of virucidal activity for instruments used in the medical area – Test method and requirements (Phase 2/Step 2).

[23] European Committee for Standardization (2015). EN 16615:2015 Chemical disinfectants and antiseptics – Quantitative test method for the evaluation of bactericidal and yeasticidal activity on non-porous surfaces with mechanical action employing wipes in the medical area (4-field test) – Test method and requirements (phase 2, step 2).

[24] Gemein S, Gebel J, Roques C, Steinhauer K, CEN/TC 216, WG 1 (2019). Practical considerations for infection prevention of near-patient surfaces: validation of an alternative polyvinyl chloride carrier in the 4-field test EN 16615:2015. J Hosp Infect.

[25] Martínez S, López M, Bernardo A (2003). Thermal inactivation of Enterococcus faecium: effect of growth temperature and physiological state of microbial cells. Lett Appl Microbiol.

[26] European Committee for Standardization EN 13727:2012+A2:2015 Chemical disinfectants and antiseptics – Quantitative suspension test for the evaluation of bactericidal activity in the medical area – Test method and requirements (Phase 2/Step 1).

[27] European Committee for Standardization (2015). EN 16616:2015 Chemical disinfectants and antiseptics – Chemical-thermal textile disinfection — Test method and requirements (phase 2, step 2).

[28] Pujato SA, Quiberoni A, Mercanti DJ (2019). Bacteriophages on dairy foods. J Appl Microbiol.

[29] European Committee for Standardization EN 13610:2002 Chemical disinfectants – Quantitative suspension test for the evaluation of virucidal activity against bacteriophages of chemical disinfectants used in food and industrial areas – Test method and requirements (phase 2, step 1). German version.

[30] Müller P, Tan CK, Ißleib U, Paßvogel L, Eilts B, Steinhauer K (2020). Investigation of the susceptibility of Candida auris and Candida albicans to chemical disinfectants using European Standards EN 13624 and EN 16615. J Hosp Infect.

[31] European Committee for Standardization (2019). EN 16777:2019 Chemical disinfectants and antiseptics – Quantitative non-porous surface test without mechanical action for the evaluation of virucidal activity of chemical disinfectants used in the medical area – Test method and requirements (Phase 2/Step 2).

[32] Steinhauer K, Meister TL, Todt D, Krawczyk A, Paßvogel L, Becker B, Paulmann D, Bischoff B, Eggers M, Pfaender S, Brill FHH, Steinmann E (2021). Virucidal efficacy of different formulations for hand and surface disinfection targeting SARS CoV-2. J Hosp Infect.

[33] Siddharta A, Pfaender S, Vielle NJ, Dijkman R, Friesland M, Becker B, Yang J, Engelmann M, Todt D, Windisch MP, Brill FH, Steinmann J, Steinmann J, Becker S, Alves MP, Pietschmann T, Eickmann M, Thiel V, Steinmann E (2017). Virucidal Activity of World Health Organization-Recommended Formulations Against Enveloped Viruses, Including Zika, Ebola, and Emerging Coronaviruses. J Infect Dis.

[34] European Committee for Standardization (2019). EN 17126:2019.

[35] Burke KE, Lamont JT (2014). Clostridium difficile infection: a worldwide disease. Gut Liver.

[36] Gemein S, Meyer B, Gebel J, Roques C, Steinhauer K, CEN TC 216, WG1 (2018). Response to J-Y Maillard: Are amine-only-containing products sporicidal?. J Hosp Infect.

[37] Reichel M, Schlicht A, Ostermeyer C, Kampf G (2014). Efficacy of surface disinfectant cleaners against emerging highly resistant gram-negative bacteria. BMC Infect Dis.

[38] Hornschuh M, Zwicker P, Kramer A, Schaufler K, Heiden SE, Bohnert JA, Becker K, Hübner NO (2021). Extensively-drug-resistant Klebsiella pneumoniae ST307 outbreak strain from north-eastern Germany does not show increased tolerance to quaternary ammonium compounds and chlorhexidine. J Hosp Infect.

[39] Stauf R, Todt D, Steinmann E, Rath PM, Gabriel H, Steinmann J, Brill FHH (2019). In-vitro activity of active ingredients of disinfectants against drug-resistant fungi. J Hosp Infect.

[40] Köhler AT, Rodloff AC, Labahn M, Reinhardt M, Truyen U, Speck S (2019). Evaluation of disinfectant efficacy against multidrug-resistant bacteria: A comprehensive analysis of different methods. Am J Infect Control.

[41] Simões M, Pereira MO, Machado I, Simões LC, Vieira MJ (2006). Comparative antibacterial potential of selected aldehyde-based biocides and surfactants against planktonic Pseudomonas fluorescens. J Ind Microbiol Biotechnol.

[42] Steinhauer K, Eschenbacher I, Radischat N, Detsch C, Niederweis M, Goroncy-Bermes P (2010). Rapid evaluation of the mycobactericidal efficacy of disinfectants in the quantitative carrier test EN 14563 by using fluorescent Mycobacterium terrae. Appl Environ Microbiol.

[43] Cadnum JL, Shaikh AA, Piedrahita CT, Sankar T, Jencson AL, Larkin EL, Ghannoum MA, Donskey CJ (2017). Effectiveness of Disinfectants Against Candida auris and Other Candida Species. Infect Control Hosp Epidemiol.

[44] Meyer B, Morin VN, Rödger HJ, Holah J, Bird C (2010). Do European Standard Disinfectant tests truly simulate in-use microbial and organic soiling conditions on food preparation surfaces?. J Appl Microbiol.

[45] European Committee for Standardization (2019). prEN 17387:2019 Chemical disinfectants and antiseptics – Quantitative non-porous surface test for the evaluation of bactericidal and/or yeasticidal and / or fungicidal activity of chemical disinfectants in the medical area – Test method and requirements without mechanical action (Phase 2/Step 1).

[46] European Committee for Standardization (2019). EN 1276:2019 Chemical disinfectants and antiseptics – Quantitative suspension test for the evaluation of bactericidal activity of chemical disinfectants and antiseptics used in food, industrial, domestic and institutional areas –Test method and requirements (Phase 2/Step 1).

[47] European Committee for Standardization EN 13697:2015+A1:2019 Chemical disinfectants and antiseptics – Quantitative non-porous surface test for the evaluation of bactericidal and / or fungicidal activity of chemical disinfectants used in food, industrial, domestic and institutional areas – Test method and requirements without mechanical action (Phase 2/Step 1).

[48] Mayfield JL, Leet T, Miller J, Mundy LM (2000). Environmental control to reduce transmission of Clostridium difficile. Clin Infect Dis.

[49] Gemein S, Gebel J, Christiansen B, Martiny H, Vossebein L, Brill FHH, Decius M, Eggers M, Koburger-Janssen T, Meckel M, Werner S, Hunsinger B, Selhorst T, Kampf G, Exner M (2019). Interlaboratory reproducibility of a test method following 4-field test methodology to evaluate the susceptibility of Clostridium difficile spores. J Hosp Infect.

[50] Eggers M, Benzinger C, Suchomel M, Hjorth E (2020). Virucidal activity of three ethanol-based hand rubs against murine norovirus in a hand hygiene clinical simulation study. Future Microbiol.

[51] European Committee for Standardization (2019). prEN 17430:2019 Chemical disinfectants and antiseptics – Hygienic handrub virucidal – Test method and requirements (phase 2/step 2).

[52] European Committee for Standardization (2019). prEN 17422:2019 Chemical disinfectants and antiseptics – Quantitative surface test for the evaluation of teat disinfectants used in the veterinary area – Test method and requirements (phase 2, step 2).

[53] European Committee for Standardization (2015). EN 14675:2015 Chemical disinfectants and antiseptics – Quantitative suspension test for the evaluation of virucidal activity of chemical disinfectants and antiseptics used in the veterinary area – Test method and requirements (phase 2, step 1).

[54] Yilmaz A, Kaleta EF (2003). Evaluation of virucidal activity of three commercial disinfectants and formic acid using bovine enterovirus type 1 (ECBO virus), mammalian orthoreovirus type 1 and bovine adenovirus type 1. Vet J.

[55] Holmes AC, Semler BL (2019). Picornaviruses and RNA Metabolism: Local and Global Effects of Infection. J Virol.

